# Gender influence on clinical manifestations, depressive symptoms and brain-derived neurotrophic factor (BDNF) serum levels in patients affected by fibromyalgia

**DOI:** 10.1007/s10067-022-06133-y

**Published:** 2022-03-28

**Authors:** Cristina Iannuccelli, Bruno Lucchino, Chiara Gioia, Giulio Dolcini, Jole Rabasco, Teresa Venditto, Francesco Ioppolo, Valter Santilli, Fabrizio Conti, Manuela Di Franco

**Affiliations:** 1 Department of Internal Clinical, Anesthesiologic and Cardiovascular Sciences, Rheumatology Unit, Sapienza University of Rome, Rome, Italy; 2 Department of Anatomical and Histological Sciences, Legal Medicine and Orthopedics, Sapienza University of Rome, Rome, Italy; 3 Unit of Physical Medicine and Rehabilitation, Umberto I Polyclinic Hospital, Sapienza University of Rome, Rome, Italy

**Keywords:** Brain-derived neurotrophic factor (BDNF), Fibromyalgia, Gender

## Abstract

**Introduction:**

**Objectives:**

Fibromyalgia (FM) is a common rheumatic disorder characterized by chronic, widespread pain associated with several not painful symptoms. The contribution of gender to the manifestation of the disease may influence the higher prevalence of FM among women. In spite of this, how patients’ gender influences the clinical manifestation of FM is still not well understood. The frequent association with neuropsychiatric symptoms raised the attention on the role of neurotrophins, including the brain-derived neurotrophic factor (BDNF) as potential biomarkers of the condition. Aims of the study were to evaluate the influence of gender on clinical manifestations and to investigate BDNF serum levels as a potential biomarker of FM.

**Methods:**

We consecutively enrolled 201 adult patients of both sexes diagnosed with FM. For each patient, we collected clinical and clinimetric data and, in a subgroup of 40 patients, we measured serum BDNF levels. BDNF levels have been measured also in 40 matched healthy controls (HC).

**Results:**

Several symptoms were significantly higher in women compared with men, including pain, fatigue, memory problems, tenderness, balance problems and sensitivity to environmental stimuli. On the contrary, men reported a significant higher frequency of coexisting depressive symptoms. BDNF levels were significantly lower in FM patients compared with HC, discriminating with good accuracy the condition.

**Conclusion:**

Gender influences FM clinical manifestations, with a higher prevalence of pain, fatigue and other common FM symptoms among women while higher frequency of neuropsychiatric symptoms among men. BDNF offers promises as a potential biomarker of the disease.

**Table:** 

**Key Points** *• Gender-related differences in the clinical manifestations of FM may contribute to the higher prevalence of FM among females. Indeed, women show higher levels of pain and symptoms traditionally associated to FM, which are evaluated to establish the diagnosis according to the clinical criteria.* *• The new insights into the pathogenesis of the disease raised the attention on the role of brain mediators in FM. Among these, BNDF shows potential as a diagnostic biomarker.*

## Introduction

Fibromyalgia (FM) is a chronic condition characterized by widespread pain, fatigue, sleep and mood disturbances and memory problems. After osteoarthritis, FM is the most common rheumatic disorder, with a prevalence varying between 2 and 8% of the general population [1]. FM has been traditionally considered an almost-exclusive women’s disorder. However, recent studies showed that, although FM is more frequent in women, the female to male ratio is 4:1 and males represent up to 40% of the overall FM patients in unbiased studies [2, 3]. The clinical picture in males and females is similar, apart from higher values of pain and symptom severity in women compared with men [3]. The presence of a gender influence in the pain severity, with higher, self-reported levels of pain by females, has been largely documented by several studies, both in healthy population and in various conditions, such as rheumatoid arthritis [4, 5]. Considering that FM diagnosis is clinical, symptoms and pain underestimation in men and overestimation women may contribute to the gender bias of FM prevalence [6]. Although the pathogenesis of the disease is still not well understood, FM is currently considered a “central sensitisation” syndrome (CSS), in which pain is the result of the activation of nociceptive pathways without clear evidence of lesions in the somatosensory system [7]. To date, there are no available biomarkers for the diagnosis of FM, which is based only on clinical features [8]. Neuropsychiatric symptoms are a strong association of FM. Indeed, up to 75% of FM patients report characteristics of axis 1 diagnosis, including anxiety or depressive disorder, suggesting a possible role of brain mediators in pathogenesis of the disease [9]. Among these, brain-derived neurotrophic factor (BDNF) is a neurotrophin functionally related to the nerve growth factor, involved in several neuroplasticity mechanisms, including growth, differentiation and repair of neurons. Although the highest level of expression has been found in the brain tissue, BDNF can pass the blood–brain barrier and can be detected in the bloodstream. Accordingly, peripheral BDNF levels are commonly used as a marker of central protein expression [10]. Abnormalities in BDNF are involved in the pathogenesis of several neuropsychiatric disorders, including anxiety and depression, as well as in stress-induced psychopathologies. Indeed, a number of studies demonstrated that peripheral BDNF levels are reduced in depressed and anxious patients, and the treatment with antidepressant is associated to an increase of BDNF levels [10–12]. BDNF is also involved in the pathogenesis of several chronic pain conditions, including FM, in which it contributes to the central sensitisation through a modulation of nociceptive stimuli and an enhanced hyperalgesia [13]. Although a number of studies investigated serum BDNF levels in FM patients, the results are conflicting, showing in some cases increased levels and in other no differences [14, 15]. The heterogeneous nature of FM itself and the presence of several confounding factors influencing BDNF serum levels, especially age and gender, may have contribute to these conflictual results [16]. Thereby, aims of our study were as follows:

- To evaluate the influence of gender on clinical manifestations, with particular regard to neuropsychiatric features of FM
- To investigate BDNF serum levels in relation to patients’ gender and clinical features
- To evaluate BDNF serum levels as a potential biomarker of FM.

## Methods

### Study design, setting and participants

In this cross-sectional study, we consecutively enrolled adult patients (18–65 years) of both sexes referring to the out-patients clinic for the diagnosis and treatment of fibromyalgia of our Policlinico Umberto I—Sapienza University of Rome, diagnosed with FM according to the 2016 revision of the 2010/2011 FM diagnostic criteria [6]. Exclusion criteria were as follows: overlap autoimmune diseases, neurologic disorders, psychiatric disorders (excluding anxiety and depression), recent use (< 3 months) of antidepressants, pregnancy. All participants provided written informed consent before their inclusion in the study. The Ethics Research Committee of the Medical Faculty, Sapienza University of Rome, approved the study, which was performed in accordance with the Helsinki Declaration.

### Patients’ assessment and clinimetric evaluation

Each participant underwent clinical evaluation performed by a rheumatologist, including the standardized assessment of 2016 diagnostic criteria and the widespread pain index (WPI) and symptoms severity scale (SSS) calculation. Moreover, also tender points examination was performed. Data regarding physical exercise, trigger events before the onset of symptoms and coexisting disorders were also collected. Each patient was asked to answer to the Italian version of the revised fibromyalgia impact questionnaire (R-FIQ) and of the Beck depression inventory-II (BDI-II) [17, 18]. R-FIQ is a validated, self-administered 20-item questionnaire, scored on a 0–10 numeric scale, designed for the evaluation of multidimensional aspects of FM. The global FIQR score defines the disease activity classification. BDI-II is a widely used 21-item self-report inventory measuring the severity of depression in adolescents and adults, scored on a 0–3 numeric scale.

### Sample size for serum experiments

Considering a confidence level of 95%, a margin error of 5%, a SD of the outcome in the population of 2.7 ng/ml and an effect size of 2.7 ng/ml, based on previous reports [19], the estimated sample size was *n* = 32. Accordingly, we enrolled 40 FM and 40 healthy control (HC) subjects.

### Sample collection and BDNF serum level evaluation

A 15-ml venous blood sample was collected from a subgroup of randomly selected, 40 enrolled patients (male to female ratio 1:1). A venous blood sample was collected also from 40 healthy volunteers, matched for age and sex, recruited from the hospital staff members as the HC group. Serum was immediately separated from blood samples through and stored at − 20 °C. After samples were defrosted, BDNF serum levels were evaluated through a commercial Double-Antibody Sandwich ELISA kit (SEA011Hu, Cloud-Clone Corp, TX, USA), following the manufacturer’s guidelines. Each sample from the same patient has been evaluated in duplicate, with a variation coefficient of 3.9%. The sensitivity for BDNF detection was 11.3 pg/ml.

### Statistical analysis

Continuous variables are shown as mean ± SD or as median (range) for normally and non-normally distributed data, respectively. Categorical variables are presented as frequencies. Comparisons of continuous variables between two groups were performed using an independent samples *T* test or Mann–Whitney *U* test, while comparisons between more than two groups were tested through the ANOVA (with Bonferroni’s correction for post hoc adjustment) or Kruskal–Wallis test, according to data distribution. Chi-squared analysis tested the differences between categorical variables. The discriminative capacity of BDNF serum levels for the presence of FM diagnosis according to the 2016 revised criteria was evaluated using ROC curves. Cut-offs with sensitivity and specificity to discriminate FM patients from healthy controls were calculated. The significance of the correlations was evaluated with Spearman’s rank correlation coefficient. All statistical analyses were performed using the SPSS Statistics version 24.0 software package (SPSS Inc., Chicago, IL, USA), and a two-sided *p* value < 0.05 was considered statistically significant.

## Results

### Overall cohort clinical and clinimetric features

The cohort was composed by a total of 201 FM patients (172 F, 29 M), mean age 49.13. Table 1 shows the clinic and clinimetric features of the patients.

**Table 1:** Clinical and clinimetric features of enrolled subjects

	Total (*n* = 201)	Females (*n* = 172)	Males (*n* = 29)	*p* value
Age (years)	49.13 (25–65)	49.07 (31–63)	49.22 (25–65)	Ns
Triggering event, %	56.2	57.4	48.2	Ns
Headache, %	80.5	82.5	68.9	Ns
Abdominal pain, %	78.6	81.9	58.6	**0**.0046*
Depressive symptoms, %	50.2	51.9	72.4	**0**.0386*
WPI	10.7 ± 4.03	10.67 ± 3.91	10.90 ± 4.81	Ns
SSS	9.17 ± 1.73	9.24 ± 1.72	8.724 ± 1.79	Ns
R-FIQ Score	**66**.**21 ± 16**.**97**	**68**.**07 ± 16**.**06**	**55**.**17 ± 18**.**26**	**0**.0005*
R-FIQ Pain	**7**.**36 ± 1**.**74**	**7**.**5 ± 1**.**64**	**6**.**52 ± 2**.**06**	**0**.0130*
R-FIQ Fatigue	**7**.**94 ± 1**.**92**	**8**.**08 ± 1**.**82**	**7**.**07 ± 2**.**25**	**0**.0141*
R-FIQ Stiffness	7.63 ± 1.99	7.74 ± 1.83	6.97 ± 2.69	Ns
R-FIQ Sleep	7.7 ± 2.33	7.76 ± 2.31	7.38 ± 2.47	Ns
R-FIQ Depression	4.9 ± 2.77	4.83 ± 2.82	5.31 ± 2.49	Ns
R-FIQ Memory	**6**.**23 ± 2**.**47**	**6**.**42 ± 2.42**	**5**.**1 ± 2.48**	**0**.0077*
R-FIQ Anxiety	6.22 ± 2.83	6.18 ± 2.89	6.45 ± 2.47	Ns
R-FIQ Tenderness	**7**.**55 ± 2**.**09**	**7**.**8 ± 1**.**95**	**6**.**07 ± 2**.**82**	** < 0**.0001*
R-FIQ Balance	**6**.**05 ± 2**.**54**	**6**.**35 ± 2**.**42**	**4**.**28 ± 2**.**52**	** < 0**.0001*
R-FIQ Env. Sensitivity	**7**.**51 ± 2**.**24**	**7**.**7 ± 2**.**10**	**6**.**41 ± 2**.**68**	**0**.0128*
BDI-II	22.88 ± 9.18	22.95 ± 9.47	22.45 ± 7.36	Ns

Data are reported as mean ± SD, unless stated otherwise. *p values intended for comparisons between female and male participants. Abbreviations: *WPI*, widespread pain index; *SSS*, symptoms severity scale; *R-FIQ*, revised fibromyalgia impact questionnaire; *BDI-II*, Beck depression inventory-II.

**Fig. 1:**
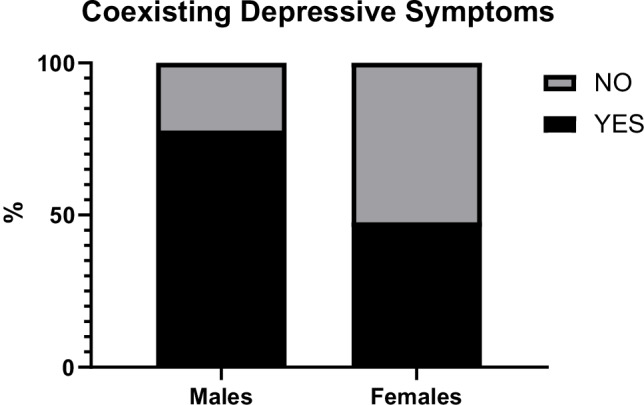
Frequency of coexisting depressive symptoms among males and females

Although no difference was found in mean WPI and SSS between males and females, women showed a significantly higher impact of symptoms in daily living in comparison with men, according to the R-FIQ score. Several items of R-FIQ were significantly higher in women compared with men, including pain, fatigue, memory problems, tenderness, balance problems and sensitivity to environmental stimuli. On the contrary, men reported a significant higher frequency of coexisting depressive symptoms compared with women, despite no difference was found in the specific R-FIQ item and in the BDI-II (Fig. 1).

### BDNF serum levels in FM patients subgroup and HC

There were no differences in terms of disease severity according to R-FIQ and BDI-II comparing the subgroup with the overall cohort. Table 2 summarizes demographic, clinical and clinimetric data and BDNF serum levels of FM patient subgroup and HC.

**Table 2:** Demographic, clinical and clinimetric features and BDNF levels of the subgroup of FM patients and HC

	FM females (*n* = 20)	FM males (*n* = 20)	HC (*n* = 40)	*p* value
Age (years)	48.6 ± 7.9	49.2 ± 10	47.6 ± 8.7	ns
BMI	24.6 ± 2.1	25.3 ± 2.9	25.2 ± 1.9	ns
BDNF, ng/ml	4.7 ± 2.4	1.8 ± 1.4	8.5 ± 3.6	** < 0**.0001*^±^
Physical exercise, %	14.2	15.7	-	ns
Disease duration, months	8.5 ± 7.7	10.3 ± 10	-	ns
Tender points	14.6 ± 3.1	11.3 ± 4.6	-	ns
WPI	10.2 ± 4.1	10.7 ± 4.9	-	ns
SSS	8.9 ± 1.6	6.3 ± 2.1	-	ns
R-FIQ TOT	62.5 ± 18.5	53.2 ± 20	-	ns
BDI-II	19.3 ± 7.4	22.6 ± 7.8	-	ns

**Fig. 2:**
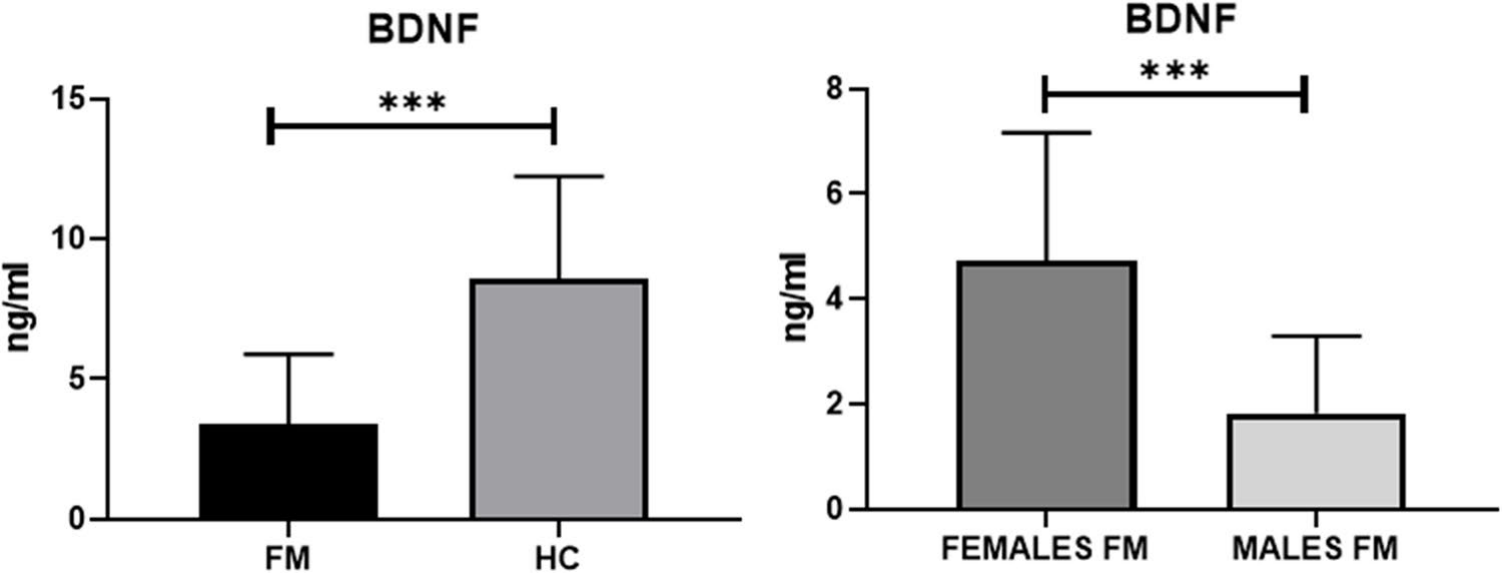
Comparison of BDNF between FM patients and HC and between female and male FM patients. ***p values < 0.0001

BDNF levels were significantly lower in FM patients compared with HC (3.38 ± 2.49 ng/dl vs 8.57 ± 3.65 ng/dl; *p* value < 0.0001). Male FM patients showed significantly lower levels of serum BDNF compared to female FM patients (4.72 ± 2.4 vs 1.82 ± 1.4. value < 0.0001) (Fig. 2). Similarly, significant lower BDNF levels were found in male FM patients compared to male HC (1.82 ± 1.4 ng/dl vs 9.03 ± 3.6 ng/dl; *p* value < 0.0001) and in female FM patients compared to female HC (4.72 ± 2.4 ng/dl vs 8.11 ± 3.7 ng/dl; *p* value = 0.0011).

**Fig. 3:**
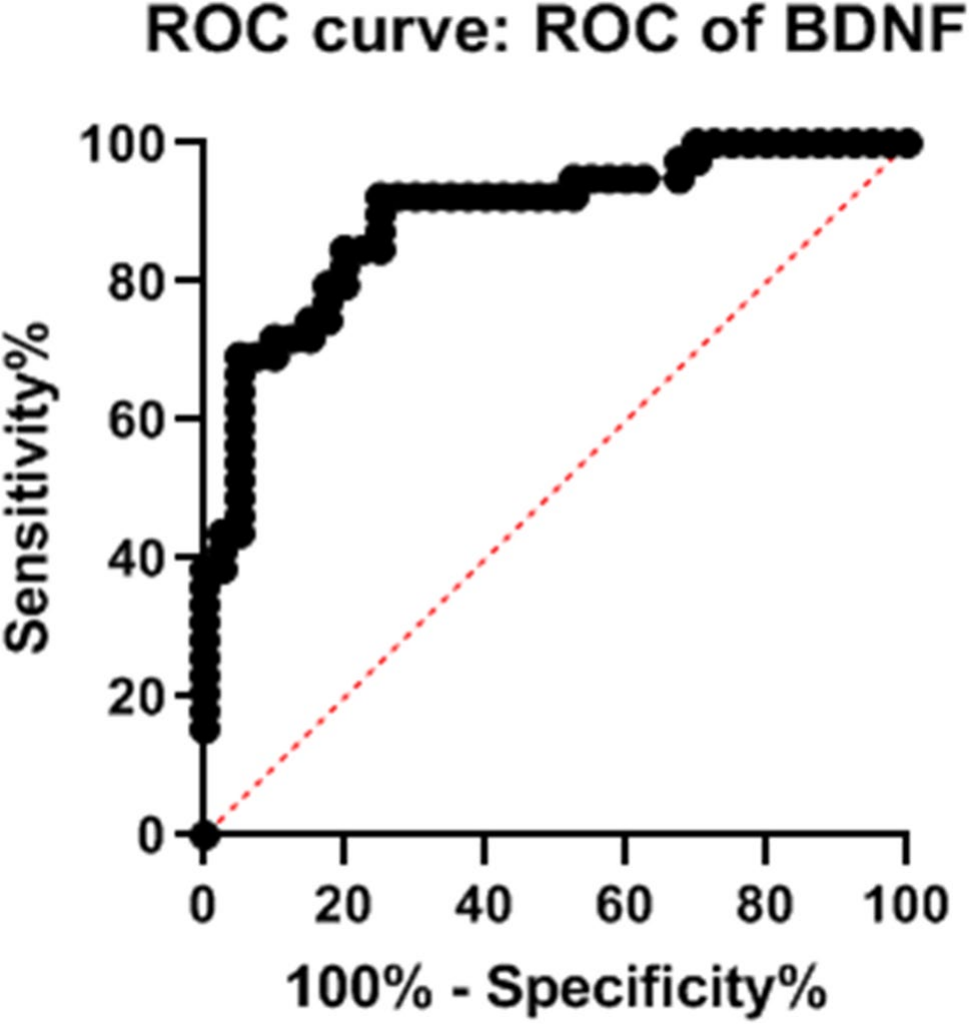
Receiver operating characteristic (ROC) curve of diagnostic performance of BDNF serum levels in the identification of FM patients

There were no relations between BDNF levels and any clinical or clinimetric feature evaluated. In order to evaluate the performance of BDNF serum levels in the identification of FM patients of both sexes with respect to HC, ROC analysis was performed (Fig. 3). The AUC value was 0.89 (95% CI: 0.82–0.96; *p* value < 0.0001). The optimal cut-off point of diagnostic performance was < 6.47 ng/ml, with a sensitivity of 92.3% and a specificity of 75% (95% CI: 79.68–97.35%) and a LR = 3.692 for identification FM patients diagnosed according the 2016 revised criteria.

## Discussion

The higher prevalence of FM in females compared with males has been confirmed by several studies, showing a female-to-male ratio of 9:1 [20]. However, more recent, unbiased studies showed that, although there is still a strong preponderance in women, the female-to-male ratio in FM is somehow lower, around 4:1, with a large underestimation of males patients affected by FM [3]. Several factors may have contribute to this discrepancy from original data. First, the social and cultural features of occidental countries, in which men are less prone to refer to a specialist for chronic pain symptoms, limiting the formulation of a correct diagnosis [21]. Moreover, the widespread belief of FM as a predominantly women’s disease, even among physicians, leading to an easier formulation of FM diagnosis in women [3]. At last, the generally higher intensity of pain symptoms in women compared to men, results in a longer window of time to formulate a diagnosis in the latter [22]. Despite a higher prevalence among men than originally thought, FM can be considered a gender-specific disease, according to not only the epidemiological differences but also to the differences in the clinical features among males and females. Indeed, in women affected by FM, higher levels of pain, fatigue, tender points count and cognitive problems as well as a higher prevalence of comorbidities, such as headache and irritable bowel syndrome, have been reported [20, 23]. Similarly, our results demonstrate higher scores in various sub-items of R-FIQ in females as well as a higher frequency of symptoms such as abdominal pain, possibly related to an irritable bowel syndrome. The global impact and the severity of the disease according to the R-FIQ global score were likewise higher in women compared to men. On contrary, there were no differences in WPI and SSS mean scores between males and females, similarly to the reported performance in the two sexes of the 2016 revised diagnostic criteria for FM [24]. Previous reports regarding the difference in the disease severity between males and females are discordant, some studies reporting a more severe disease among men while others showing no differences [25, 26]. The major limitation of these studies, which is besides present in our study, is the small number of male patients evaluated, narrowing a reliable assessment of the difference. However, this limit was present also in the validation study of the R-FIQ itself, which the psychometric properties are less reliable in males affected by FM [17]. Accordingly, two major considerations can be made: the higher self-reported pain by female patients, and the major role of pain-related symptoms in the diagnosis of FM according to 1990 criteria could represent a source of bias in the actual definition of FM, especially in male patients. Indeed, the increasing use of the revised 2016 diagnostic criteria, more evenly performant in the two sexes, can limit this issue, providing study populations more faithful to the clinical picture [3]. Moreover, our study suggests that several differences in the clinical manifestations of FM in the two sexes exist. Accordingly, taking into account the limit of R-FIQ psychometric performance in males, a gender-specific instrument for disease severity assessment in FM could be desirable and could better explore the symptomatic differences of affected patients. Data regarding gender differences in coexistent depression among FM patients are controversial. The Nord–Trøndelag Health Study suggested a greater role of anxiety in females and a greater role of depression in males affected by FM [27]. In a recent, large, online-based survey, men affected by FM reported depression over both widespread pain and sleep disturbances [28]. On the other hand, other studies reported no differences in the prevalence of depression between the two sexes [26]. Our results suggest a significant higher prevalence of depressive symptoms in males. As mentioned above, the majority of the available literature about the gender difference in FM are based on the 1990 definition, which underestimates male patients and their clinical features. Newer studies are needed to evaluate the importance and frequency of depression in males with FM, diagnosed according the revised 2016 criteria.

Although 2016 diagnostic criteria represents a step forward, the lack of biomarkers for the diagnosis of FM is still a major issue in clinical practice. BDNF is diffusely expressed across the nociceptive pathway in the central nervous system (CNS), where it can influence pain perception through the modulation of synaptic plasticity. Although several studies indicate a strong involvement in the nociceptive system, the role of BDNF is still uncertain, with some studies showing a pro-nociceptive while other studies show an anti-nociceptive effects [29]. The deprivation of neurotrophic effect of BDNF has been involved in an increased inflammatory cytokines release and the neurodegeneration associated with Alzheimer disease, while the protective effect of the physical exercise on memory loss has been related to an increased BDNF production [30, 31]. The neurotrophic effects of BDNF have been particularly studied in depression. A reduced expression of BDNF in various brain areas, including the hippocampus and prefrontal cortex, has been described in depressed patients, and can be reversed by treatment with various anti-depressants. Depressed patients show reduced serum levels of BDNF compared with healthy subjects [32]. Several brain regions involved in depression are shared with CSS, and BDNF exerts a complex role in the physiopathology of these conditions. While an increased production of BDNF can enhance and maintain hyperalgesia at peripheral and spinal level, on the other hand, the global effect of BDNF in brain tissue is more uncertain. Recent evidence suggests that neuro-inflammation and microglial activation in specific areas of brain are central in the pathogenesis of CSS, especially FM, while the peripheral abnormalities in the small sensory fibres are not associated with any significant somatosensory dysfunction [33, 34]. Microglial cytokine production may represent the main mechanism of BDNF suppression, in a similar fashion as demonstrated in major depression. Several products of activated microglia, such as inteleukin-1, nitric oxide and prostaglandins have been shown to suppress the expression of BDNF and are related to depressive symptoms, cognitive disorders and fatigue [32, 35, 36]. The reduction of the neuroprotective function of BDNF may in part explain the association with these clinical features, shared between depression and FM. Recent studies showed that the presence of the val66met BDNF gene polymorphism, a genetic variant associated with a reduced production of BDNF, is associated to an increased susceptibility of chronic pain disorders and, in FM patients, to pain catastrophizing and central sensitisation symptoms [37, 38]. Our results show reduced serum levels of BDNF in FM patients, contrarily to previous reports of increased or indifferent levels [14, 15]. Although several factors may have contribute to this discrepancy, a full explanation cannot be provided. Despite the available studies measured BDNF using either serum and plasma interchangeably, plasma and serum BDNF reflect two different pools with different biological relevance, and plasma BDNF levels are susceptible of a greater variability [39]. BDNF levels are also influenced by age and physical exercise, which were not significantly different between males and females in our cohort [16, 39]. Moreover, a potential bias in drawing definitive conclusions was the limited number of patients included in BDNF evaluation, despite there were no apparent disease severity differences compared to the overall cohort. Serum BDNF levels need to be evaluated in larger cohorts in order to fully assess the biomarker potential in FM identification. To the best of our knowledge, this is the first study investigating serum BDNF levels in patients affected by FM diagnosed according to 2016 revised criteria. Overcoming the diagnosis mainly based on tender points examination and consequently increasing the number of patients with not-painful symptoms classified as having FM may have affected the measurement of BDNF serum levels, especially considering the role of BDNF reduction on mood and cognitive symptoms. The increasing understanding of neuro-inflammation and neurotrophic factor role in CSS may in future increase our knowledge about the variation of BDNF peripheral levels. However, the diagnostic accuracy of BDNF measurement showed by our study for FM identification according to the newer criteria suggests further studies to investigate a potential clinical utility.

## Conclusion

Although the gender discrepancy in FM has been a largely investigated subject of research, the application of the newest diagnostic criteria led to a better definition of clinical FM and substantially changed both the epidemiologic and clinical scenarios, increasing the number of males classified as having FM. However, several differences in the clinical manifestations between the sexes still exist and R-FIQ psychometric properties may underestimate symptoms in males affected by FM. Serum BDNF measurement showed a good accuracy in FM patient identification. Considering the increasing knowledge about the role of BDNF in FM, larger studies are needed to confirm its clinical utility and to evaluate a potential association mainly with not painful symptoms.
